# MATCHER: manifold alignment reveals correspondence between single cell transcriptome and epigenome dynamics

**DOI:** 10.1186/s13059-017-1269-0

**Published:** 2017-07-24

**Authors:** Joshua D. Welch, Alexander J. Hartemink, Jan F. Prins

**Affiliations:** 1 Department of Computer Science, The University of North Carolina at Chapel Hill, Chapel Hill, NC USA; 2 Curriculum in Bioinformatics and Computational Biology, The University of North Carolina at Chapel Hill, Chapel Hill, NC USA; 3 Department of Computer Science, Duke University, Durham, NC USA

**Keywords:** Single cell RNA-seq, Single cell epigenomics, Manifold learning, Manifold alignment

## Abstract

Single cell experimental techniques reveal transcriptomic and epigenetic heterogeneity among cells, but how these are related is unclear. We present MATCHER, an approach for integrating multiple types of single cell measurements. MATCHER uses manifold alignment to infer single cell multi-omic profiles from transcriptomic and epigenetic measurements performed on different cells of the same type. Using scM&T-seq and sc-GEM data, we confirm that MATCHER accurately predicts true single cell correlations between DNA methylation and gene expression without using known cell correspondences. MATCHER also reveals new insights into the dynamic interplay between the transcriptome and epigenome in single embryonic stem cells and induced pluripotent stem cells.

**Electronic supplementary material:**

The online version of this article (doi:10.1186/s13059-017-1269-0) contains supplementary material, which is available to authorized users.

## Background

Understanding the mechanisms that regulate gene expression across space and time is a fundamental challenge in biology. Epigenetic modifications such as DNA methylation, histone marks, and chromatin accessibility are known to regulate gene expression, but the precise details of this regulation are not well understood. Single cell genomic technologies reveal heterogeneity within populations of cells, including complex tissues, tumors, and cells undergoing temporal changes [1, 2]. Furthermore, because bulk data consist of measurements averaged across a population of cells, single cell genomic data enable, in principle, much more precise study of how epigenetic changes and gene expression vary together.

Single cell RNA-sequencing (RNA-seq) has been applied with great success to the study of sequential cellular processes such as differentiation and reprogramming [3–7]. In such experiments, each sequenced cell is assumed to be at one point in the process and sequencing enough cells can reveal the progression of gene expression changes that occur during the process [8, 9]. More recently, several experimental techniques for performing single cell epigenetic measurements have been developed [10–17] and several studies have demonstrated that single cell epigenetic data can be also used to elucidate the series of changes in a sequential process [16, 18, 19].

Identifying correlations among epigenome and transcriptome dynamics would allow more complete understanding of the sequential changes that cells undergo during biological processes. Measuring multiple genomic quantities from a single cell, or multi-omic profiling [20, 21], would be the best way to identify such correlations. Unfortunately, performing single cell multi-omic profiling is very difficult experimentally, because an assay on chromatin or RNA destroys the respective molecules and only tiny amounts of DNA and RNA are present in a single cell. In certain cases, it is possible to assay RNA and DNA [14, 22–24] or RNA and proteins [25, 26] from the same single cell, but experimentally performing multiple assays on either chromatin or RNA from the same cell is extremely challenging.

Our knowledge of epigenetic regulation suggests that any large changes in gene expression, such as those that occur during differentiation, are accompanied by epigenetic changes. Therefore, it should be possible, in principle, to infer sequential changes in cellular epigenetic state during a process. Furthermore, if cells undergoing a common process are sequenced using multiple genomic techniques, examining any of the genomic quantities should reveal the same underlying biological process. For example, the main difference among cells undergoing differentiation will be the extent of their differentiation progress, whether you look at the gene expression profiles or the chromatin accessibility profiles of the cells.

We reasoned that this property of single cell data could be used to infer correspondence between different types of single cell measurements. To infer single cell correspondences, we use a technique called manifold alignment [27, 28]. Intuitively, manifold alignment constructs a low-dimensional representation (manifold) for each of the observed data types, then projects these representations into a common space (alignment) in which measurements of different types are directly comparable. To the best of our knowledge, manifold alignment has never been used in genomics. However, other application areas recognize the technique as a powerful tool for multimodal data fusion, such as retrieving images based on a text description, and multilingual search without direct translation [28]. We refer to our method as MATCHER (Manifold Alignment to CHaracterize Experimental Relationships). Using MATCHER, we identified correlations between transcriptomic and epigenetic changes in single mouse embryonic stem cells (mESCs) and single human induced pluripotent stem cells (iPSCs).

## Results and discussion

### Overview of MATCHER

Manifold alignment is an approach for integrating multiple types of data that describe different aspects of a common phenomenon. For example, a video of a person speaking, an audio recording of the speech, and a written transcript of the words uttered all describe a common set of events from different perspectives. The key idea of manifold alignment, as initially proposed by Ham et al. [29], is to integrate multiple data types by discovering the common manifold structure that underlies them. In many real-world settings, the assumption of a common underlying manifold generating multiple data types is a reasonable one. There are two main types of manifold alignment, distinguished by whether they require examples of precisely corresponding measurements to align manifolds (manifold alignment with correspondence) [29] or simply use geometric information (manifold alignment without correspondence) [30]. Gaussian process latent variable models have also been used to perform manifold alignment (with correspondence) by learning completely [31, 32] or partially [31] shared latent representations of high-dimensional, multimodal data. Given a set of images and corresponding text descriptions, manifold alignment can be used to identify a low-dimensional representation that allows the prediction of a caption for a new image. This is somewhat analogous to the problem of retrieving a corresponding epigenetic measurement for a given single cell transcriptome. However, in the context of single cell genomic data, correspondence information is not generally available to train a model, because it is very difficult to measure more than one quantity on a single cell. Therefore, we developed a novel approach for manifold alignment without correspondence that leverages the unique aspects of this problem.

We assume that:

1. Single cell genomic data from cells proceeding through a biological process lie along a one-dimensional manifold. Another way of saying this is that the variation among cells can be explained mainly by a single latent variable (“pseudotime”) corresponding to position within the process.
2. Each of the genomic quantities under consideration changes in response to the same underlying process.
3. The biological process is monotonic, meaning that progress occurs only in one direction. Processes that alternate between forward and backward progress or repeat cyclically would violate this assumption.
4. The cells in each experiment are sampled from the same population, process, and cell type.

**Fig. 1:**
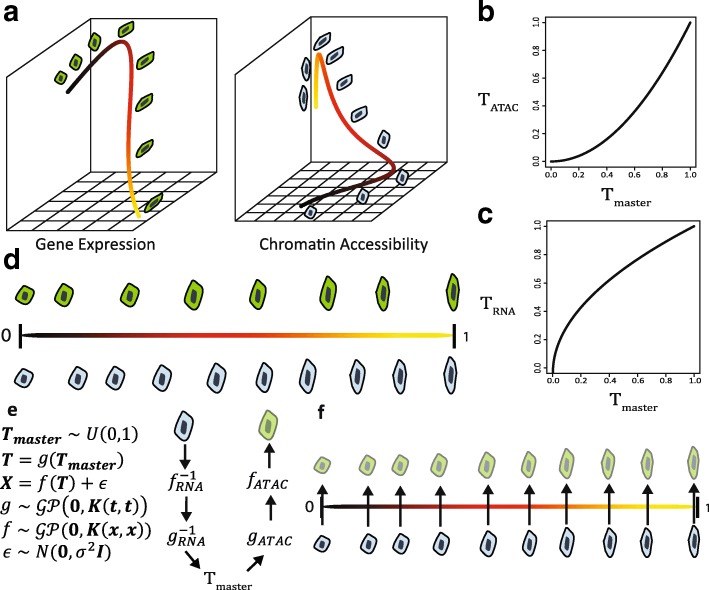
MATCHER method overview. **a** We infer manifold representations of each dataset using a Gaussian process latent variable model (GPLVM). However, the resulting “pseudotime” values from different genomic data types are not directly comparable due to differences in orientation, scale, and “time warping.” Both the *color* of the *curve* (*black* to *yellow*) and cell morphology (blob to oblong) indicate position within this hypothetical process. **b**, **c** To account for these effects, pseudotime for each kind of data is modeled as a non-linear function (warping function) of master time using a Gaussian process. **d** MATCHER infers “master time” in which the steps of a biological process correspond to values uniformly distributed between 0 and 1 and are comparable among different data types. However, different datasets are measured from different physical cells and thus may sample different points in the biological process and even different numbers of cells. **e** *Diagram* showing how MATCHER’s generative model can infer corresponding cell measurements. The generated cell is drawn with transparency to indicate that this is an inferred rather than observed quantity. **f** Applying MATCHER to multiple types of data provides exactly corresponding measurements from observed cells and unobserved cells (indicated with transparency) generated by MATCHER

Given these assumptions, there are only three possible types of differences among the one-dimensional manifold representations of each data type: orientation; scale; and “time warping” (Fig. 1a). We can perform manifold alignment without correspondence information by accounting for these three types of differences. Differences in orientation can occur if the biological process corresponds to increasing manifold coordinates for one type of genomic data but decreasing coordinates for another data type. We can reconcile different orientations by simply reversing the order of one set of manifold coordinates. It is not possible to infer the correct orientation from data, so we rely on biological prior knowledge to choose the correct orientation for the manifold inferred from each type of data. To address scale differences, we can normalize the manifold coordinates to lie between 0 and 1. Time warping effects can occur if different genomic quantities change at different rates. For example, gene expression changes may occur slowly at the beginning of a process and gradually speed up, while changes in chromatin accessibility may show exactly the opposite trend during the process (Fig. 1a). We account for time warping effects by learning a monotonic warping function for each type of data (see below for details).

We use a Gaussian process latent variable model (GPLVM) to infer pseudotime values separately for each type of data. A GPLVM is a non-linear, probabilistic, generative dimensionality reduction technique that models high-dimensional observations as a function of one or more latent variables [33]. The key property of a GPLVM is that the generating function is a Gaussian process, which allows Bayesian inference of latent variables non-linearly related to the high-dimensional observations [34, 35]. The non-linear nature of this model makes it more flexible than a technique such as principal component analysis (PCA) that uses a linear model. In fact, PCA can be derived as a special case of a GPLVM in which the Gaussian process generating function uses a linear kernel [33]. Importantly, GPLVMs are also generative models, meaning that they can answer the counterfactual question of what an unobserved high-dimensional data point at a certain location on a manifold *would* look like. The generative nature of GPLVMs is particularly important to our approach: we use this property to infer correspondence among single cell genomic quantities measured in different ways. We note that GPLVMs have previously been used to infer latent variables underlying differences among single cell gene expression profiles [36–38]; our approach differs from these previous approaches in that we use GPLVMs as part of a *manifold alignment* approach and *generate* measurements from unobserved cells to *integrate* multiple types of single cell measurements.

After inferring pseudotime separately for each type of data, we learn a monotonic warping function (Fig. 1b, c) that maps pseudotime values to “master time” values, which are uniformly distributed between 0 and 1 (Fig. 1d). This is equivalent to aligning the quantiles of the pseudotime distribution to match the quantiles of a uniform random variable. Master time values inferred from different data types are then directly comparable, corresponding to the same points in the underlying biological process.

The model that we use to infer master time values (Fig. 1e) allows us to *generate* corresponding cell measurements even from datasets where the measurements were performed on different single cells. The different types of measurements may produce datasets with cells from different positions in the biological process and even different numbers of cells (Fig. 1e). To generate a corresponding measurement for a cell, we take the master time value inferred for a given cell, such as one measured with RNA-seq. Then we map this master time value through the warping function to a pseudotime value for a different type of data, such as ATAC-sequencing (ATAC-seq). Using the GPLVM trained on ATAC-seq data, we can output a corresponding cell based on this pseudotime value. As Fig. 1f shows, the generative nature of the model allows MATCHER to infer what unobserved cells measured with one experimental technique *would* look like if they corresponded exactly to the cells measured using a different technique. These corresponding cell measurements can then be used in a variety of ways, such as computing correlation between gene expression and chromatin accessibility.

Although it is very difficult in general to measure multiple genomic quantities on the same single cell, two protocols, scM&T-seq [14] and sc-GEM [39], have been developed for measuring DNA methylation and gene expression in the same single cell. It is possible that future protocols will enable other joint measurements. In such cases, MATCHER can perform manifold alignment with correspondence using a shared GPLVM [40] to infer a shared pseudotime latent variable for both data types (see below for details).

MATCHER takes as input multiple types of single cell measurements performed on cells of the same type, but not necessarily the exact same cells. Each type of data is provided to MATCHER as a matrix, where rows correspond to cells and columns correspond to features. MATCHER outputs master time values for each cell and inferred corresponding measurements.

### Data description and processing

Several high-throughput single cell versions of epigenetic assays have been developed, including single cell bisulfite sequencing (DNA methylation) [14], ATAC-seq (chromatin accessibility) [13], and chromatin immunoprecipitation sequencing (ChIP-seq) (histone modification) [12]. Each of the initial studies that pioneered these methods applied them to mESCs grown in serum, a classic model system of stem cell biology. Cells in this system are heterogeneous, differing depending on where they are located along a spectrum ranging from a pluripotent ground state to a differentiation primed state [41]. Note that mESCs grown in serum have different properties than mESCs cultured in 2i medium, which are much more homogeneous and differ primarily in their cell cycle stage [36, 41].

We also analyzed single cell gene expression and DNA methylation data generated by sc-GEM [39], a protocol that measures DNA methylation and gene expression in the same cells, from human cells undergoing reprogramming to iPSCs.

We collected the publicly available data from these papers. In total, we have four kinds of single cell data from a total of 5151 cells: 250 cells with gene expression data only [41], 238 with DNA methylation and gene expression [14, 39], 76 with chromatin accessibility [13], and 4587 with H3K4me2 ChIP [12].

The processing of single cell epigenetic data is more difficult than RNA-seq, because the epigenetic data are nearly binary at each genomic position (apart from allele-specific effects and copy number variations) and extremely sparse, with only a few thousand reads per cell in many cases. This makes it very difficult to extract any meaningful information at base pair resolution from a single cell. Instead, we followed the data processing steps laid out in each of the respective papers that developed these techniques and aggregated the reads across related genomic intervals (see “Methods” for details). For example, we followed the authors’ lead in summing the chromatin accessibility data values from ATAC-seq in a given cell across all of the binding sites for a given transcription factor. Doing this for each of 186 transcription factors results in a matrix of 186 chromatin accessibility signatures across the set of cells. The DNA methylation data and H3K4me2 ChIP-seq data were aggregated in a similar way. We obtained the processed DNA methylation and ChIP-seq data from the initial publications. The processed ATAC-seq data are not publicly available, so we processed the data by implementing ourselves the pipeline described in the paper. We found that the proportion of relevant events (methylated CpGs, accessible chromatin sites, or histone modifications) captured per cell was the highest for the DNA methylation data; the ChIP-seq data were the sparsest. Consequently, it was sufficient to aggregate the DNA methylation data over relatively small genomic intervals such as individual promoters or CpG islands.

### Single cell transcriptome and epigenome data show common modes of variation

It seems likely that gene expression, DNA methylation, chromatin accessibility, and histone modifications will all change during the transition from pluripotency to a differentiation primed state. However, we wanted to investigate that this crucial assumption holds in this particular system.

**Fig. 2:**
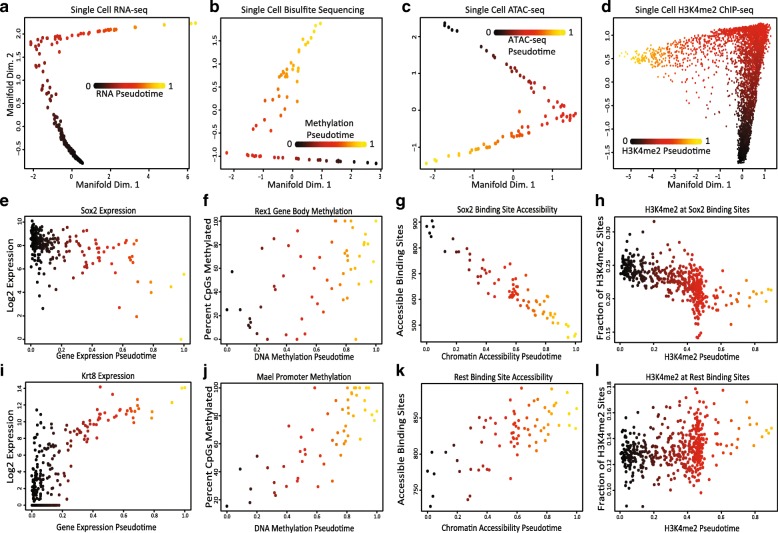
Single cell transcriptome and epigenome data show common modes of variation. **a**–**d** Single cell trajectories constructed by SLICER from RNA-seq, bisulfite sequencing, ATAC-seq, and H3K4me2 ChIP-seq of mESCs grown in serum. **e**–**l** Levels of important gene expression, DNA methylation, chromatin accessibility, and H3K4me2 markers across the trajectories. Note: We used SLICER for the analysis in this figure because it is a previously published method for constructing cell trajectories that allowed us to investigate the hypothesis that single cell transcriptome and epigenome measurements share common sources of variation. SLICER and MATCHER are completely separate methods; MATCHER does not rely on SLICER in any way; and SLICER could not be used to integrate multiple types of measurements as MATCHER does, because SLICER lacks the ability to generate unobserved cell measurements

To test our hypothesis that each of these epigenetic data types are changing over the course of a common underlying process, we first attempted to construct a cell trajectory for each type of data. Using SLICER, a method we previously developed [9], we visualized each type of data as a two-dimensional (2D) projection and inferred a one-dimensional ordering for the cells. The 2D projections show that each type of data resembles a one-dimensional trajectory rather than a 2D blob of points (Fig. 2a–d). Note that these 2D projections do not force the data into a one-dimensional shape; the plots could look like a diffuse point cloud and the fact that they instead resemble trajectories shows that the differences can be explained by a single latent variable. Furthermore, the projections of each kind of data are strikingly similar visually (Fig. 2a–d).

We further investigated these trajectories to determine whether they correspond to the same underlying process. The trajectory built from RNA data shows decreasing expression of pluripotency genes such as SOX2, consistent with previously published analyses [41] (Fig. 2e). DNA methylation of the gene body of *Rex1*, a gene that is shut off during the transition from pluripotency to differentiation priming [42], increases during the process (Fig. 2f). The single cell ATAC-seq data show that the chromatin accessibility of binding sites for the SOX2 transcription factor decreases over pseudotime (Fig. 2g). Similarly, the levels of H3K4me2, a histone modification associated with active enhancers and promoters, decrease at SOX2 binding sites (Fig. 2h). The RNA-seq data show increasing expression of previously identified differentiation markers [41] such as *Krt8* (Fig. 2i). DNA methylation of the promoter for *Mael* increases, consistent with previous findings [42] (Fig. 2j). Both the chromatin accessibility (Fig. 2k) and H3K4me2 levels (Fig. 2l) at REST binding sites increase, consistent with the known role of REST in repressing key lineage-specifying genes [43, 44]. In summary, our analysis indicates that each type of single cell data varies along a trajectory, establishing a continuum that ranges from pluripotency to a differentiation primed state.

We used SLICER to perform this initial exploratory analysis, but for the rest of this study, we use MATCHER, which is completely separate from SLICER and does not rely on the method in any way. We did confirm, however, that the master time values inferred by MATCHER are highly correlated with the pseudotime values inferred by SLICER (Additional file 1: Figure S1). Note also that SLICER cannot be used to integrate multiple types of single cell measurements in the way the MATCHER does, because the model underlying SLICER is not generative.

### MATCHER accurately models synthetic and real data

To evaluate the accuracy of MATCHER, we generated synthetic data for which ground truth master time is known. We generated data by sampling 100 master time values uniformly at random from the interval [0, 1], then mapping these to pseudotime values through a warping function. Using the resulting pseudotime values, we generated 600 “genes,” each following a slightly different “expression pattern” (function of pseudotime). Normally distributed noise was added to each gene expression value. We then used MATCHER to infer master time from these simulated gene expression values and measured accuracy as the correlation between true and inferred master time values. Note that we use Pearson rather than Spearman correlation because we expect true and inferred master time to be linearly related (equal, in fact), and a non-linear relationship would indicate that the inference process is inaccurate. The results of our simulations indicate that MATCHER accurately infers master time across a range of different warping functions and noise levels (Additional file 1: Figures S2 and S3). The method is very robust to noise in the simulated genes, yielding a correlation of 0.92 at a noise level of *σ* = 9, which is greater than 50% of the range of the simulated features.

We also tested MATCHER on real data. We used scM&T-seq data, in which DNA methylation and gene expression are measured in the same single cells [14], so that the true correspondence between single cell measurements is known. Note that we used the known cell correspondence information for validation only, not during the inference process; we are using the correspondence information provided by scM&T-seq as a gold standard and the method does not require such information. We first checked the relationship between master time inferred by MATCHER from RNA-seq and DNA methylation data by calculating the correlation between inferred master time values for corresponding DNA methylation and RNA-seq cells. This showed that the master time values, although not identical, are highly concordant (Pearson *ρ* = 0.63).

**Fig. 3:**
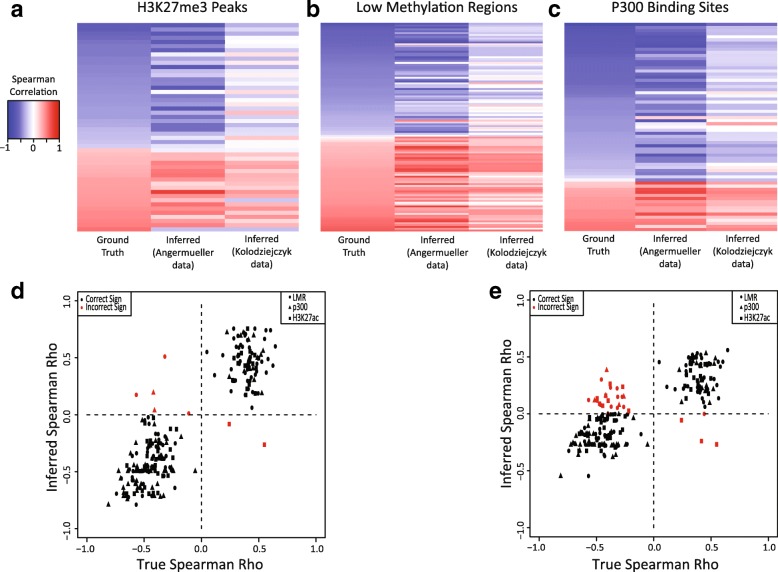
MATCHER accurately infers known correlations between DNA methylation and gene expression. **a**–**c** *Heatmaps* comparing true correlations between gene expression and DNA methylation of related regions (H3K27me3 peaks, LMRs, and P300 binding sites). The first *column* of each heatmap shows the true correlation based on known correspondence information, the second column shows the correlation inferred by MATCHER in the same dataset, and the third column is correlation inferred by MATCHER using a completely different single cell RNA-seq dataset from mESCs grown in serum. **d**, **e** *Scatterplot* representation of the results shown in (**a**–**c**). **d** Correlations computed using the Angermueller data. **e** Correlations computed from the Kolodziejczyk data. Each *point* represents the true and inferred correlation for a single gene-site pair; ideal results would lie along the y = x line. Note that the sign of the inferred correlation is correct for the vast majority of pairs

Predicting covariance of multiple genomic quantities across single cells is one of the key applications of MATCHER. Therefore, as an additional test, we investigated whether MATCHER can accurately infer correlations between DNA methylation events and gene expression. Here, we used Spearman correlation because we are interested in both linear and non-linear relationships. We selected a set of genes and proximal methylated loci that showed statistically significant correlation in the original analysis of the scM&T-seq data [14]. Angermueller et al. grouped these pairs according to the type of region where the methylation site occurred. We selected the three types of regions with the largest number of significant pairs (low methylation regions, H3K27me3 peaks, and P300 binding sites). Then, for each significant pair, we compared the true correlation (calculated using true cell correspondences) and correlation inferred by MATCHER (calculated using inferred cell correspondences). We also used MATCHER to compute correlations for the same gene-locus pairs using a single cell RNA-seq dataset published by a different lab [41]. In this dataset, the cells measured using RNA-seq are the same cell type, but not the same physical cells as those assayed for DNA methylation by Angermueller et al. In both cases, the inferred correlations closely match the true correlations (Fig. 3). The mean absolute deviation between true and observed correlations in the Angermueller dataset is 0.16. The correlations computed using the Kolodziejczyk data show slightly less concordance with the ground truth (mean absolute deviation = 0.27), likely due to the inevitable biological and technical variation that occur when different labs repeat an experiment. Even so, the vast majority of inferred correlations have the correct sign and the relative magnitude of correlations tends to be preserved.

### Correlations among single cell gene expression, chromatin accessibility, and histone modifications

**Fig. 4:**
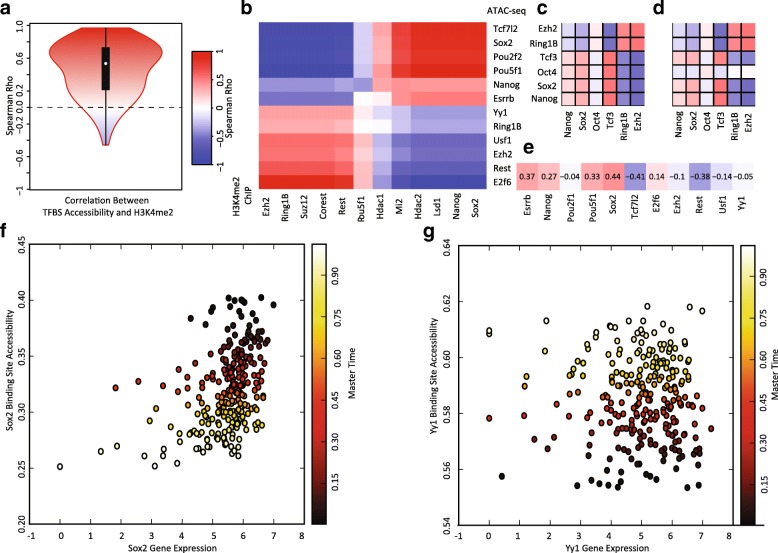
Correlations among single cell gene expression, chromatin accessibility, and histone modifications. **a** *Violin plot* of correlations among chromatin accessibility and H3K4me2 of transcription factor binding sites for 186 transcription factors. Note that most correlations are strongly positive. **b** Correlation between chromatin accessibility and H3K4me2 data reveals that targets of pluripotency factors/NuRD complex and targets of Polycomb Group/Trithorax Group proteins are anticorrelated in single cells. **c** Correlation between gene expression signatures and chromatin accessibility signatures. **d** Correlation between gene expression signatures and H3K4me2 signatures. **e** Correlation between gene expression of DNA binding proteins and chromatin accessibility of their targets. **f** Inferred corresponding values of *Sox2* gene expression and chromatin accessibility of SOX2 binding sites. Each *point* represents inferred correspondence from a single cell. The *x-axis* shows the value of the gene expression signature in that cell and the *y-axis* shows the value of the chromatin accessibility signature. The points are colored by inferred master time. **g** Inferred corresponding values of *Yy1* gene expression and chromatin accessibility of YY1 binding sites

We next used MATCHER to investigate the relationships among gene expression, chromatin accessibility, and histone modifications during the transition from pluripotency to a differentiation primed state in mESCs. To our knowledge, this is the first time that investigation of the relationship among these three genomic quantities has been performed in single cells. We performed this analysis with two primary goals: (1) to confirm that the correlations among gene expression, chromatin accessibility, and H3K4me2 agree with what is known from bulk analysis (Fig. 4a, c); and (2) to demonstrate some of the unique insights that can be derived by correlating these quantities across individual cells (Fig. 4b, d–f). All of the correlation analyses described below are computed by taking the vector of values for a gene or set of genomic regions (such as binding sites for SOX2) across a set of single cells and correlating this vector with the values for another gene or set of genomic regions (such as binding sites for OCT4) across the set of single cells. Because the gene expression, chromatin accessibility, and H3K4me2 measurements that we are analyzing were performed on different single cells, this analysis is possible only because of MATCHER’s ability to infer corresponding measurements. In summary, although some of the results that we describe recapitulate previous results from analysis of bulk data, all of our analyses here are novel in that correlations are computed *across individual cells within a heterogeneous population*.

As an initial sanity check, we tested whether H3K4me2 and chromatin accessibility values within corresponding sets of genomic regions are positively correlated across the set of single cells (Fig. 4a). Because H3K4me2 is a histone modification associated with promoter and enhancer activation, we expect levels of the modification to correlate positively with chromatin accessibility. We confirmed this is, indeed, the case by inferring correlations between chromatin accessibility and H3K4me2 at the respective regions bound by 186 transcription factors and DNA binding proteins. For example, we correlated the chromatin accessibility at SOX2 binding sites across cells with the H3K4me2 levels at SOX2 binding sites across cells. The vast majority of these correlations are positive, consistent with previous findings from bulk data and with the role of H3K4me2 as an activating chromatin mark.

While investigating the correlation between H3K4me2 and chromatin accessibility, we found that the genomic binding regions clustered into two main groups: (1) pluripotency transcription factors and the NuRD complex; and (2) chromatin remodeling factors that repress or activate lineage specific genes (Fig. 4b). Rotem et al. noted a similar relationship in the H3K4me2 data [12]. The accessibility of binding sites for OCT4 (also known as POU5F1), NANOG, and SOX2, well-established pluripotency transcription factors, is strongly anticorrelated with the accessibility of binding sites for EZH2, RING1B, and SUZ12, which are Polycomb Group (PcG) proteins [45]. The targets of the transcription factor YY1, which recruits PcG proteins [46], show a similar trend to the PcG proteins. Given that PcG proteins play a key role in repressing neuronal lineage genes in pluripotent cells [47], this anticorrelation suggests that chromatin is being remodeled to prime lineage-specific genes while shutting down regions associated with pluripotency. REST and COREST show a similar pattern to the PcG proteins; these proteins are known to co-associate with the polycomb repressive complex (PRC2) and also to repress key lineage specific genes in pluripotent cells [43, 44]. Interestingly, the targets of USF1, which is known to recruit Trithorax Group (TrxG) proteins [48], also show a pattern of increasing chromatin accessibility. The TrxG proteins are chromatin activators that regulate lineage differentiation genes [47–49], suggesting that the activation of certain differentiation genes is occurring while their repression by PRC2 is being lifted. Finally, targets of LSD1, MI2, HDAC1, and HDAC2, components of the NuRD complex, show positive correlation with targets of pluripotency factors. The NuRD complex contains chromatin remodeling proteins that remove histone methylation and histone acetylation marks and function to “decommission” pluripotency enhancers during early differentiation [50]. In summary, our analysis of correlation between chromatin accessibility and H3K4me2 marks indicate that the overall trend in both types of data is toward chromatin changes that shut off pluripotency and begin to lift lineage repression in preparation for differentiation.

As an additional sanity check, we investigated whether chromatin accessibility and H3K4me2 are positively correlated with the expression of genes within the corresponding regions. For this analysis, we chose to focus specifically on the binding regions for EZH2, RING1B, TCF3, OCT4, SOX2, and NANOG. Because of the way we aggregated genomic regions when analyzing chromatin accessibility and ChIP-seq data, we needed a comparable way to aggregate the expression of genes within these regions. After locating genes whose promoters overlapped each of these binding regions, we filtered the sets of genes to remove genes that occurred in multiple binding regions. We then normalized the expression of each gene (zero mean, unit variance) and calculated the aggregate expression within each cell for each set of genes. These aggregate expression levels of genes whose promoters occur within the binding regions of each of the six proteins are then directly comparable with the chromatin accessibility and H3K4me2 from the same set of binding regions within each cell. Note again that we are correlating these quantities across single cells—each cell has six aggregate expression values and corresponding chromatin accessibility and H3K4me2 values. As expected, the aggregate expression of these sets of genes correlates well with the chromatin accessibility and H3K4me2 of the gene promoters (Fig. 4c, d), with the exception of OCT4. The expression of OCT4 targets are only weakly correlated with the aggregate chromatin accessibility and H3K4me2. Additional file 1: Figures S4 and S5 show the corresponding values inferred by MATCHER for gene expression, chromatin accessibility, and H3K4me2 values in the same single cells.

To demonstrate that MATCHER can reveal unique insights not possible with bulk data, we investigated how the gene expression levels of key pluripotency factors and chromatin remodeling proteins correlate with the chromatin accessibility of their binding sites during the transition from naïve to primed pluripotency (Fig. 4e–g). In this analysis, we made use of the fact that MATCHER tells us both (1) the relationship between chromatin accessibility and gene expression in individual cells and (2) the trends of both of these quantities over master time. This allowed us to begin to tease apart how different regulatory mechanisms—both chromatin and expression—operate during a sequential biological process. Using the same transcription factors and DNA binding proteins as in Fig. 4b, we inferred corresponding expression levels for each gene and the overall chromatin accessibility of the sites where its protein product binds to the genome (Fig. 4e). For example, we correlated the vector of expression levels for the *Sox2* gene across the set of single cells with the vector of chromatin accessibility for the targets of the SOX2 protein across the set of cells. Note that we are looking at the accessibility of the *targets* of these DNA binding proteins, not the *promoters* of the genes that encode these factors (although, in some cases, a protein may target the promoter of the gene that encodes it).

The pluripotency transcription factors ESRRB, NANOG, POU5F1, and SOX2 each show positive correlation between expression and chromatin accessibility, with both expression and chromatin accessibility showing an overall decreasing trend over master time (Fig. 4e). Figure 4f shows the corresponding gene expression and chromatin accessibility values inferred for SOX2 and their relationship with master time. This indicates that the expression of these genes is being shut off at the RNA level at the same time as the binding of the factors is shut off at the chromatin level. Interestingly, *Tcf7l2* gene expression shows strong negative correlation with the chromatin accessibility of its targets. We speculate that this negative correlation may be due to the fact that TCF7L2 functions primarily as a transcriptional repressor [51] and thus increased expression will lead to more repression of its targets.

In contrast to the pluripotency factors, the expression of genes involved in chromatin remodeling show weak negative correlation with the accessibility of their binding sites (Fig. 4e). The chromatin accessibility of these factors’ targets shows an increasing trend over master time, but the expression of the chromatin remodeling factors does not vary significantly over master time. The inferred corresponding values for Yy1 are shown as an example in Fig. 4g. Thus, changes in the chromatin accessibility of the targets of these chromatin remodeling complexes occurs without accompanying changes in the gene expression levels of the remodelers, indicating that regulation is occurring primarily at the chromatin level in this case. The one exception is the *Rest* gene, whose expression decreases over master time and shows strong negative correlation with the accessibility of its binding sites.

To understand the advantages of using MATCHER in this way to analyze a combination of omics data from single cells, it is instructive to imagine a comparable bulk experiment and what insights it might yield. One could perform bulk RNA-seq, ATAC-seq, and ChIP-seq on separate populations of ESCs. However, the cellular differences that we have observed here occur among stem cells grown in a common culture environment. A comparable bulk analysis would require some sort of purification (FACS, MACS, etc.) to isolate populations of naïve and primed cells grown in serum. Even if such populations were purified, they would likely still contain a mixture of cells at various points on the spectrum from ground state to primed pluripotency. Furthermore, such an experiment would allow only “early” and “late” comparisons, rather than examination of the continuous trends that MATCHER provides. Consequently, one could identify genes with higher population expression in ground state versus primed cells and regions of chromatin that are generally more accessible in ground state versus primed cells, but not any of the intermediate changes in expression or chromatin that occur during the transition from ground state to primed pluripotency. The point of this discussion is not to disparage bulk sequencing experiments, which are extremely useful, but rather to argue that there is also a place for the sort of integrative single cell multi-omic analysis that we performed here. We believe that, just as trajectory analysis of single cell RNA-seq data has proven useful for studying many important biological processes, MATCHER will reveal novel biology when applied to future single cell transcriptomic and epigenomic data.

### Relationship between DNA methylation and gene expression during transition from ground state to primed pluripotency

**Fig. 5:**
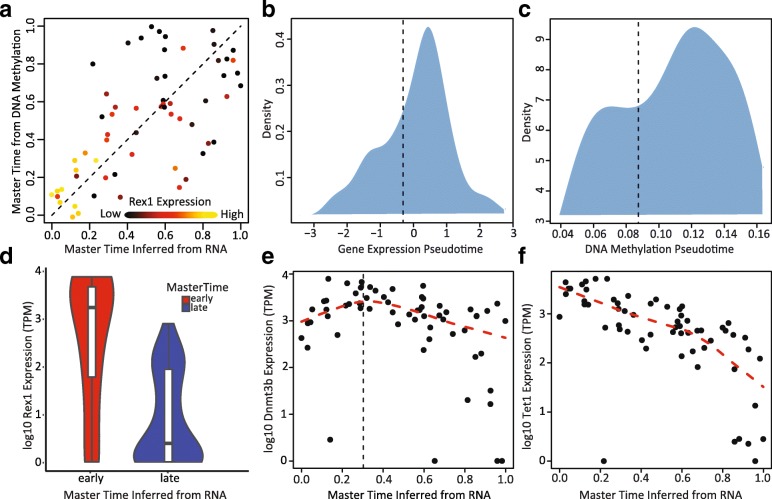
Relationship between gene expression and DNA methylation in transition from ground state to primed pluripotency. **a** *Scatterplot* showing the relationship between master time inferred from gene expression and master time inferred from DNA methylation. Points are colored by the log10 expression of *Rex1*. The *dotted line* is the y = x line. Note that the gene expression and DNA methylation master time values are more correlated before master time = 0.3 than after. **b**, **c** *Density plots* showing the distribution of pseudotime inferred from **b** gene expression and **c** DNA methylation. The *vertical dotted line* indicates the 30th percentile of pseudotime (master time = 0.3). **d** *Violin plot* showing the distribution of Rex1 expression in cells before master time = 0.3 (“early”) and after master time = 0.3 (“late”). **e** Expression of *Dnmt3b* as a function of gene expression master time. The *red line* is a LOESS smoothing function indicating the overall expression trend. The *black vertical line* indicates master time = 0.3. **f** Expression of *Tet1* as a function of gene expression master time. The *red line* is a LOESS smoothing function indicating the overall expression trend

We next used MATCHER to investigate the interplay between gene expression and DNA methylation in mESCs. We first examined the relationship between master time inferred from gene expression and master time inferred for the same cells using DNA methylation (Fig. 5a). (Note that here we are using the known correspondences available from scM&T-seq to compare master time values inferred separately from DNA methylation and gene expression for identical cells.) This analysis showed an intriguing relationship: DNA methylation and gene expression master time track together quite well until a specific point in gene expression master time, around master time = 0.3. After that point, the degree of coupling suddenly decreases. This result is consistent with the results of the initial analysis of the scM&T-seq data, which found variability in the strength of coupling between gene expression and DNA methylation across the set of cells [14].

To assess the significance of the apparent partial decoupling between DNA methylation and gene expression, we computed separate Pearson correlation values for cells with gene expression master time less than 0.3 and greater than 0.3. Then we performed Fisher’s *r*-to-*z* transformation on the correlations and computed a *p* value for the null hypothesis that the correlation before master time = 0.3 is less than or equal to the correlation after master time = 0.3 (one-tailed test). The *p* value was 0.037, indicating a significant difference at *p* = 0.05. We also performed a permutation test, in which we sampled (without repetition) a random division of the cells into two groups consisting of approximately 30% and 70% of cells, calculated the Spearman correlation between the gene expression and DNA methylation master time values in the two groups separately, and subtracted the two correlation values. Repeating this sampling procedure 100,000 times gave an empirical *p* value of 0.0025 for the null hypothesis that the correlation before master time = 0.3 is less than or equal to the correlation after master time = 0.3. We also confirmed that both analyses are robust to the choice of division point in master time: the difference in correlations is also significant (*p* < 0.05) if master time = 0.5 is used as the dividing line.

We hypothesized that the observed relationship may occur because specific *de novo* DNA methylation changes are required to trigger a key step in the process of gene expression changes during the transition from ground state pluripotency to a primed state, but after this point in the process, the sequential gene expression changes proceed somewhat independently from the DNA methylation changes. A previous single cell study of mESCs grown in serum showed the existence of two metastable expression states, corresponding to ground state and primed pluripotency [42]. The *Rex1* gene was previously shown to be a marker for these metastable expression states, with high *Rex1* expression in the ground state and low *Rex1* expression in the primed state [42]. Singer et al. also found that the transition between these two states is dependent on the activity of DNA methyltransferase (DNMT) enzymes, and knocking out DNMT activity greatly increases the proportion of cells in the *Rex1*-high state [42].

In support of this hypothesis, the cells in which DNA methylation and gene expression correlate strongly show high levels of *Rex1* expression, while the remaining cells show much lower expression (Fig. 5a and d). We also found that the distributions of pseudotime values for both gene expression and DNA methylation are highly non-uniform and roughly bimodal (Fig. 5b and c). This pattern is consistent with the existence of two metastable states, suggesting that cells tend to accumulate toward the beginning and end of pseudotime and transition fairly rapidly in between. In further support of this model, the two modes of the distribution account for approximately 30% and 70% of cells, respectively (Fig. 5b and c); these proportions correspond to the divergence point (master time = 0.3) noted in Fig. 5a.

To further investigate the potential role of *de novo* methylation in the transition from the ground state to the primed state, we examined the expression trends of *Dnmt3b*, a gene encoding a DNMT, and *Tet1*, a gene implicated in demethylation (Fig. 5e and f). Singer et al. previously found the expression of these two genes to be strongly negatively and positively correlated with *Rex1* expression, respectively [42]. Intriguingly, we find that *Dnmt3b* shows a transient pulse of expression, with initially increasing expression that peaks, then steadily decreases (Fig. 5e). The peak of Dnmt3b expression occurs precisely at master time = 0.3, which fits well with the data in Fig. 5a–d and is also consistent with a model in which *de novo* methylation activity increases to help cells escape the *Rex1*-high state. *Tet1* expression is highest at the beginning of master time and steadily decreases (Fig. 5f). These two observations together suggest that *Tet1* actively maintains low methylation levels in the *Rex1*-high state but is gradually downregulated while a pulse of *Dnmt3b* expression occurs, leading to the accumulation of methylation and transition to the *Rex1*-low state. These results also suggest that *de novo* methylation is required primarily to transition away from the *Rex1*-high state, and both *de novo* methylation activity and demethylation gradually subside after this transition, stabilizing the DNA methylation profiles of the cells.

It is worth noting that the partial decoupling we have just described is not the same as complete decoupling. The master time values that MATCHER inferred separately from DNA methylation and gene expression are highly correlated (*p* = 0.63), and our results shows that the method accurately predicts the ground truth correlations between DNA methylation and gene expression in single cells (mean absolute deviation of 0.16). We have chosen to use the term “partial decoupling” to indicate that DNA methylation and gene expression are somewhat, but not completely, predictive of each other. MATCHER does not require that the measurements be completely coupled and our analysis here shows that the method still performs well even in the presence of partial decoupling. It is perhaps not surprising that DNA methylation and gene expression do not perfectly predict each other, because gene expression is regulated by many factors in addition to DNA methylation. Our discovery of this partial decoupling does highlight the fact that simultaneous experimental measurements, such as scM&T-seq, provide additional information that MATCHER cannot infer. Nevertheless, MATCHER provides a useful tool for analyzing single cell transcriptomic and epigenomic data, whether or not experimentally determined cell correspondences are available.

### Analysis of gene expression and DNA methylation changes during human iPSC reprogramming

We used MATCHER to analyze data from sc-GEM, a protocol (distinct from scM&T-seq) that allows simultaneous measurement of pre-selected DNA methylation and gene expression markers in single cells using polymerase chain reaction (PCR) [39]. Cheow et al. performed sc-GEM on human fibroblasts undergoing iPS cell reprogramming. Unlike the mESC data that we analyzed above, the Cheow dataset contains multiple time points, from 0 to 24 days after the start of the reprogramming process. We downloaded the processed, normalized PCR data from the Cheow paper, and did not perform additional processing.

**Fig. 6:**
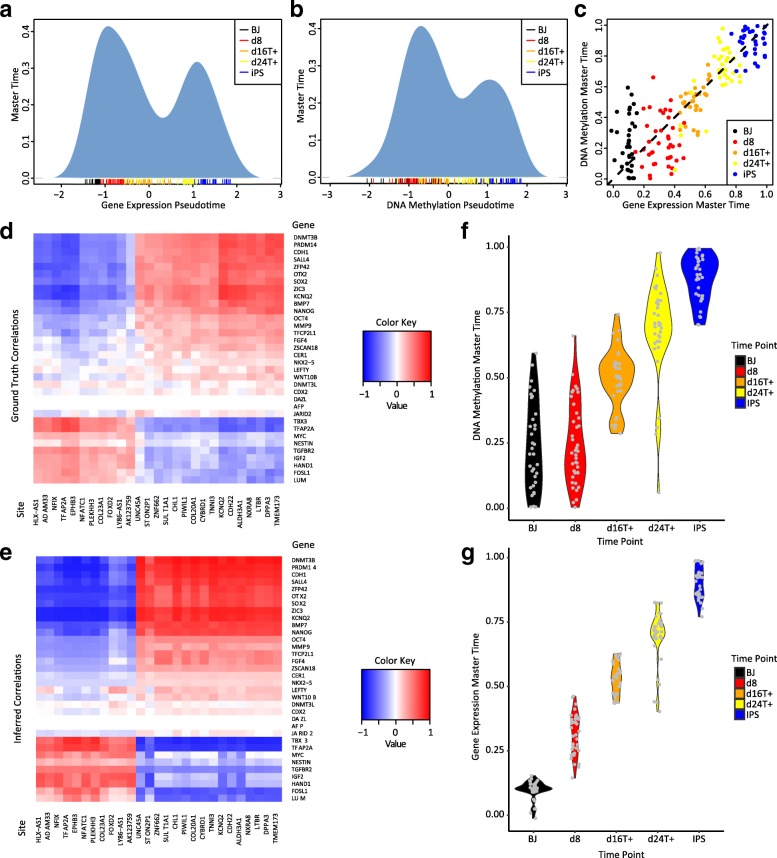
Analysis of gene expression and DNA methylation in human fibroblast cells undergoing reprogramming. **a**, **b** *Density plots* showing distribution of pseudotime inferred from **a** gene expression and **b** DNA methylation. The pseudotime values for individual cells are shown as a *rug plot* below the density plot; *color* indicates the time point. **c** Relationship between master time inferred from gene expression and master time inferred from DNA methylation. **d** *Heatmap* of ground truth correlation between expression of all genes measured in the sc-GEM experiment and DNA methylation level of all promoters measured. **e** *Heatmap* of correlation inferred by MATCHER from sc-GEM data. Note that MATCHER inferred these correlations without using the known correspondence among cells in any way. **f** *Violin plot* of the DNA methylation master time values for cells at each time point. Note that the distributions for untreated fibroblasts (BJ) and fibroblasts eight days after treatment (d8) are virtually identical. **g** *Violin plot* of the gene expression master time values for cells at each time point

When we used MATCHER to analyze the Cheow data, we found that, as with the mESCs, the distribution of pseudotime inferred from both DNA methylation and gene expression was bimodal rather than uniform (Fig. 6a and b). This pattern suggests that only unprogrammed fibroblast cells and successfully reprogrammed iPSCs are stable; cells transitioning between states are relatively unstable and thus transition relatively rapidly. Unlike in the case of ESCs, DNA methylation and gene expression master time values appear to be strongly correlated throughout the entire iPS reprogramming process (Pearson *ρ* = 0.89; see Fig. 6c).

Because sc-GEM provides measurements where the true correspondence between cells and correlation between DNA methylation and gene expression are known, this dataset provides an additional opportunity to assess the accuracy of MATCHER. To do this, we computed the true Spearman correlation between all pairs of genes and promoters assayed in the sc-GEM experiment. Then, we compared these true values to the values inferred by MATCHER. As with the scM&T-seq dataset described above, MATCHER’s inferred correlations closely matched the true values (Fig. 6d and e), with a mean absolute deviation of 0.17.

The experimental design of the Cheow dataset, which contains multiple time points, allows us to utilize both temporal and pseudotemporal information. We therefore investigated whether we could use the time point information to learn anything about the relative ordering of DNA methylation and gene expression changes. Our analysis suggests that DNA methylation changes lag behind gene expression changes. As Fig. 6f shows, the day 0 (BJ) fibroblasts and day 8 fibroblasts span nearly identical portions of master time inferred from DNA methylation and signs of reprogramming are apparent only at day 16 or beyond. In contrast, gene expression master time shows a continual, steady progression, with only a handful of cells overlapping the master time range of the previous time point (Fig. 6g). Thus, enough gene expression changes occur within eight days of the reprogramming process to distinguish untreated cells and day 8 cells, but it takes longer than eight days for distinguishing DNA methylation changes to occur. In other words, the gene expression changes occur temporally prior to the DNA methylation changes. Consistent with this result, the relative height of the iPSC mode in Fig. 6b is less than the relative height of the iPSC mode in Fig. 6a, indicating that fewer cells have moved beyond the DNA methylation profile of the starting fibroblast state than have moved beyond the starting gene expression state. We note that sc-GEM experiment measured only a pre-selected subset of genes and promoters, so we cannot rule out the possibility that the DNA methylation status of other genomic loci could distinguish the untreated and day 8 fibroblasts. Nevertheless, our findings are consistent with a previous report that the vast majority of the DNA methylation changes in iPS reprogramming occur after day 9 [52].

One of the motivations for developing MATCHER was to enable integration of single cell datasets in which cells do not exactly correspond. Therefore, we performed additional analysis to demonstrate that, even though sc-GEM provides measurements from exactly corresponding cells, MATCHER does not require this information. To simulate datasets in which DNA methylation and gene expression were measured separately on distinct cells of the same type, we repeatedly sampled a random 75% or 50% of sc-GEM gene expression profiles and a random 75% or 50% of sc-GEM DNA methylation profiles. This analysis showed that we could reproduce the results in Fig. 6 using a dataset without exactly corresponding cells (Additional file 1: Figure S6).

Finally, we note that the lagging behavior observed here does not violate the assumptions of MATCHER; in fact, this is an example of just the sort of “time warping” behavior that is shown in the hypothetical example of Fig. 1a. Comparing the master time ranges for corresponding time points in Fig. 6f and g shows that the warping functions inferred by MATCHER are largely able to correct for this effect. For example, days 0–8 span the same master time range for both DNA methylation and gene expression. If MATCHER did not correct for time warping, day 8 DNA methylation measurements would be matched only with day 0 gene expression cells; day 16 DNA methylation cells would be matched only with day 8 gene expression measurements; and so on.

### Incorporating known cell correspondence information to infer shared master time

So far, we have used MATCHER to infer separate master time values for each type of transcriptomic or epigenomic measurement. Our results demonstrate that such an approach can reveal important insights, whether the true cell correspondences are known or unknown. However, in cases where multiple measurements are performed simultaneously on the same cells, as with scM&T-seq and sc-GEM, it could also be informative to infer a shared cell ordering that indicates each cell’s overall progress in terms of both transcriptomic and epigenomic changes. We now demonstrate how to infer “shared master time” using MATCHER and give an example of how such analysis can be useful.

To infer shared master time, MATCHER uses a shared GPLVM [40] to infer pseudotime in place of a separate GPLVM for each data type. The shared GPLVM assumes that each type of measurement is generated, through different mappings, from a common (“shared”) latent space [40]. After inferring pseudotime using a shared GPLVM, MATCHER uses Gaussian process regression to learn a warping function and infer master time values that are uniformly distributed between 0 and 1, in the same way as when pseudotime values are inferred separately for each data type.

**Fig. 7:**
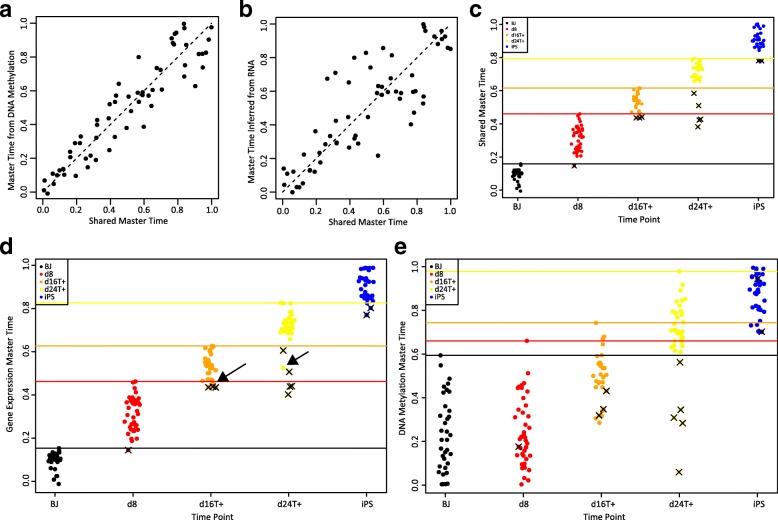
Incorporating known cell correspondence information to compute shared master time. **a** *Scatterplot* of shared master time inferred from both gene expression and DNA methylation (*x-axis*) and master time inferred using DNA methylation only (*y-axis*). **b** *Scatterplot* of shared master time inferred from both gene expression and DNA methylation (*x-axis*) and master time inferred using gene expression only (*y-axis*). **c** *Plot* showing “lagging cells” whose shared master time values overlap with the master time values of a previous time point. The x-values are jittered to mitigate overplotting. *Colored horizontal lines* indicate the maximum master time value for the corresponding time point. Lagging cells are indicated by “*x*” symbols. **d** *Plot* showing differences between lagging cells identified from shared master time and lagging cells identified from gene expression master time alone. The “*x*” symbols indicate lagging cells identified using shared master time. *Arrows* indicate two cells that are lagging based on gene expression master time alone but not shared master time. **e** *Plot* showing differences between lagging cells identified from shared master time and lagging cells identified from DNA methylation master time alone. The “*x*” symbols indicate lagging cells identified using shared master time

We first used MATCHER to infer a shared master time value using both DNA methylation and gene expression data for each cell assayed with scM&T-seq (Fig. 7a and b). The resulting shared master time values reconcile the sequence of changes occurring in both genomic quantities. The Pearson correlation between DNA methylation master time and RNA master time is 0.63. In contrast, the correlation between DNA methylation master time and shared master time is 0.93 (Fig. 7a); the correlation between RNA master time and shared master time is 0.84 (Fig. 7b).

We also inferred shared master time for cells assayed with sc-GEM (Fig. 7c–e). As an example of how this shared master time can be used, we identified “lagging cells” whose shared master time values overlap with the shared master time values of cells from an earlier time point (Fig. 7c). These cells lag behind other cells from the same time point in terms of both their gene expression and DNA methylation reprogramming progress. Using either gene expression (Fig. 7d) or DNA methylation (Fig. 7e) alone to identify lagging cells gives conflicting sets of cells; some cells whose gene expression lags show timely methylation changes and vice versa. Thus, it is not clear which of these cells should be considered lagging in the overall process of reprogramming both DNA methylation and gene expression. Shared master time provides a principled way to reconcile the two perspectives obtained from gene expression and DNA methylation measurements and determine the overall reprogramming progress of each cell.

## Conclusion

In this study, we used MATCHER to characterize the corresponding transcriptomic and epigenetic changes in ESCs undergoing the transition from pluripotency to a differentiation primed state and iPSCs undergoing reprogramming. Interesting future directions of research include extending the model to align manifolds with dimensionality higher than one, as well as adapting the method for cell populations whose cells fall into discrete clusters rather than along one continuous spectrum. In addition, our model does not explicitly account for branching trajectories, which can arise in biological processes with multiple outcomes [3, 9]. A simple way to handle such situations would be to assign cells to branches before running MATCHER, and then perform manifold alignment on each branch separately.

Although the Hi-C protocol for measuring chromatin conformation has been adapted to single cells [10], we did not include single cell Hi-C data in this study for two reasons. First, to the best of our knowledge, there are no published single cell Hi-C datasets from the cell types that we investigated. In addition, Hi-C data are a set of pairwise interactions (a matrix for each cell rather than a vector), and it is not clear how to construct a trajectory from this type of data. Further work is necessary to investigate whether chromatin conformation shows sequential changes during biological processes, as well as the best ways infer such sequential changes and integrate them with other types of data.

One promising application of the method is aggregating single cell measurements into biologically meaningful groups. Cells can be grouped by their inferred master time values, and measurements within these groups can be aggregated. In experiments with thousands of cells, this will likely enable correlation between individual loci and related genes, which is currently impossible because of the extreme sparsity of the epigenetic data. Computational aggregation of measurements from many similar single cells may be the most immediate way to address the sparsity of single cell epigenetic measurements, although experimental protocols will likely improve over the long term.

MATCHER gives insight into the sequential changes of genomic information, allowing the use of both single cell gene expression and epigenetic data in the construction of cell trajectories. In addition, it reveals the connections among these changes, enabling investigation of gene regulatory mechanisms at single cell resolution. MATCHER promises to be useful for studying a variety of biological processes, such as differentiation, reprogramming, immune cell activation, and tumorigenesis.

## Methods

### RNA-seq data processing

We obtained the processed RNA-seq data for 250 cells from Kolodziejczyk et al. [41]. In the original paper, gene quantification was performed using read counts that were normalized for sequencing depth and batch effects [41]. We log transformed these normalized counts and used our previously published neighborhood variance method to select an informative subset of genes to feed into MATCHER.

To identify populations of RNA molecules with a clear relationship to the aggregated genomic regions used to compute chromatin accessibility and histone modification measurements (see below), we computed analogous aggregated gene expression measurements. We did this by identifying genes whose promoters overlap binding sites for each of six proteins (EZH2, RING1B, TCF3, OCT4, SOX2, and NANOG). We then filtered the gene lists so that a given gene appears on only one of the six lists. Then we scaled and centered each gene to have zero mean and unit variance and computed the sum of the genes on each list per cell, as well as the total sum of expressed genes in each cell. The final values used to compute correlations shown in Fig. 4c and d are the centered and scaled differences of the sum for each list of genes and the total sum of gene expression per cell; we refer to these values as gene expression signatures.

### ATAC-seq data processing

The processed single cell ATAC-seq data are not publicly available, so we implemented the data processing pipeline described by Buenrostro et al. [13] For each cell, we aligned reads to mm10 using bowtie2, removed PCR duplicates, and counted the number of reads aligning to each of the 50,000 peaks identified in the initial paper [13]. We converted these integer read counts, which are predominantly 1 or 0 at a given peak, into binary values (1 for accessible chromatin, 0 for inaccessible) to avoid potential confounding factors that could cause high counts such as copy number variations and repeat elements. Then we used FIMO [53] to identify, for each peak, which of the 186 transcription factor motifs in the JASPAR database [54] occurs in the peak region. Using this peak-to-TF mapping, we aggregated the peak counts for each cell by summing the peaks for each transcription factor motif. This gave a matrix with 186 features across 96 cells. We subsequently removed all cells with fewer than 1000 peaks detected per cell, leaving 77 cells. Dimensionality reduction using PCA and a GPLVM on the 77 cells indicated that one cell was a significant outlier, so we removed this additional cell. The remaining 76 cells were used for all subsequent analyses. We then normalized the 186 × 76 matrix to account for differences among cells in numbers of peaks detected. We normalized the value of *f*_*ij*_ (feature *i* in cell *j*) by multiplying by the following scale factor: ${s}_{ij}=\left({\sum}_j{t}_j/n\right)/\left(1000\cdot {t}_j\right)$, where *t*_*j*_ is the total number of accessible peaks in cell *j*. (The 1000 in the denominator of the scale factor scales the measurements so that the *f*_*ij*_ are close to 1.)

### ChIP-seq data processing

We obtained the processed data from Rotem et al. [12], which consists of H3K4me2 ChIP-seq reads from 4587 cells, aggregated using 91 chromatin signatures. We found that these data required further normalization for the total sum of signature values per cell. We normalized the value of *f*_*ij*_ (signature *i* in cell *j*) by multiplying by the following scale factor: ${s}_{ij}=10\cdot \left(\sum_j{t}_j/n\right)/{t}_j$, where *t*_*j*_ is the total sum of signatures in cell *j*. (The 10 in the numerator of the scale factor scales the measurements so that the *f*_*ij*_ are close to 1).

### scM&T-seq data processing

RNA-seq and DNA methylation data from Angermueller et al. [14] are publicly available in fully processed form, so we did not perform any further processing. In the original paper, the gene expression levels were computed by counting unique molecular identifiers (UMIs) and subsequently normalized. The DNA methylation values from Angermueller were also normalized in the original paper [14].

We initially tried using the methylation values from all positions in the genome, but PCA and GPLVM results on the full dataset showed no systematic variation related to pluripotency and differentiation. This is likely because only a subset of methylation sites shows systematic biological variation in excess of technical variation during the transition from pluripotency to differentiation priming. We therefore selected methylation sites based on a previously validated marker, *Mael*, whose methylation is known to change during the transition to a differentiation primed state [42]. We selected all methylation sites whose correlation with the promoter methylation of *Mael* was at least 0.2. This gave a set of approximately 13,000 methylation sites. There were essentially no methylation sites anticorrelated with *Mael*, consistent with the fact that pluripotent cells are globally demethylated, so that methylation changes in preparation for differentiation occur primarily in a single direction. We also found that using only data from low methylation regions (LMRs), which are known to change methylation state dramatically during differentiation, gives similar results [55].

### Inferring pseudotime

We infer pseudotime using a Gaussian process latent variable model (GPLVM) with a single latent variable ***t***. For a more thorough introduction to Gaussian processes and GPLVMs, see Rasmussen [56] or Damianou [35]. Under our model, the observed high-dimensional data (RNA-seq, ATAC-seq, ChIP-seq, DNA methylation, etc.) are generated from ***t*** by a function *f* with the addition of Gaussian noise:

$$ \boldsymbol{Y}=f\left(\boldsymbol{t}\right) + \boldsymbol{\epsilon} $$

where $$ \boldsymbol{\epsilon} \sim \mathcal{N}\kern0.28em \left(0,{\sigma}^2\boldsymbol{I}\right) $$.

The key property of a GPLVM is that the prior distribution of *f* is a Gaussian process:

$$ f\left(\boldsymbol{t}\right) \sim \mathcal{G}\mathcal{P}\left(0,k\left(\boldsymbol{t},{\boldsymbol{t}}^{\mathbf{\hbox{'}}}\right)\right) $$

A linear kernel yields a model equivalent to probabilistic PCA, but if we choose the kernel function *k* to be non-linear, the GPLVM can infer non-linear relationships between *t* and *Y*. We use the popular radial basis function (RBF) kernel, also called the squared exponential kernel.

$$ k\left({t}_i,{t}_j\right)\kern0.5em =\kern0.5em {\sigma}_{rbf}^2 \exp \left(-\frac{1}{2{l}^2}{\left({t}_i-{t}_j\right)}^2\right) $$

Because a Gaussian process is a collection of random variables for which the covariance of any finite set is a multivariate Gaussian, we have:

$$ P\left(Y\Big|\boldsymbol{t},{\sigma}^2,{\sigma}_{rbf}^2,l\right) = \mathcal{N}\left(Y\Big|0,{K}_{ff}+{\sigma}^2\boldsymbol{I}\right) $$

where *K*_*ff*_ is the covariance matrix defined by the kernel function *k*. A simple approach to inferring the latent variable ***t*** would be to find the values that maximize the posterior distribution:

$$ {\boldsymbol{t}}_{\boldsymbol{MAP}} = \arg \underset{\boldsymbol{t}}{ \max }P\left(\boldsymbol{Y}\Big|\boldsymbol{t}\right)P\left(\boldsymbol{t}\right) $$

Instead of MAP estimation, we use the method of Damianou [35], which estimates the posterior using a variational approximation. A key advantage of this approach is that it provides a distributional estimate of the latent variables rather than just a point estimate. The approximation relies on the introduction of auxiliary variables called inducing inputs to derive an analytical lower bound on the marginal likelihood. Inference is then performed by maximizing the lower bound with respect to the inducing inputs and the hyperparameters *σ* ^2^, *σ*_*rbf*_^2^, and *l*. We used ten inducing inputs for all of our analyses, although we confirmed that the results are robust to the number of inducing inputs used. We used the Bayesian GPLVM model implemented in the GPy package, with the default initialization setting, which uses PCA to determine the initial values for the latent space before optimization.

To infer shared master time from simultaneous measurements (such as scM&T-seq or sc-GEM), we first use a shared GPLVM [40] to infer pseudotime, then proceed to infer a warping function in the same way as for pseudotime values inferred from a regular GPLVM (see next section for details). The shared GPLVM model extends the regular GPLVM by assuming that multiple types of high-dimensional data (such as gene expression and DNA methylation measurements) ***Y*** ^(1)^, ***Y*** ^(2)^ are generated from a shared latent space through different mapping functions:

$$ \begin{array}{l}{\boldsymbol{Y}}^{(1)} = {f}_1\left(\boldsymbol{t}\right) + \kern0.5em {\upepsilon}_1\\ {}{\boldsymbol{Y}}^{(2)} = {f}_2\left(\boldsymbol{t}\right) + \kern0.5em {\upepsilon}_2\end{array} $$

As with the regular GPLVM, we used an RBF kernel *k* to calculate covariance among points in the latent space; however, for the shared GPLVM, each data type has a separate set of hyperparameters *σ* ^2^, *σ*_*rbf*_^2^, and *l*. The shared GPLVM model is a special case of a more general technique called manifold relevance determination, in which latent dimensions can be weighted differently in the covariance function for each data type [31]. The manifold relevance determination model uses an automatic relevance determination (ARD) kernel with a separate weight for each latent dimension. For example, for the RBF automatic relevance determination kernel is:

$$ k\left({\boldsymbol{t}}_i,{\boldsymbol{t}}_j\right)\kern0.5em =\kern0.5em {\sigma}_{rbf}^2 \exp \left(-\frac{1}{2}{\displaystyle \sum_k}{w}_k{\left({t}_{ik}-{t}_{jk}\right)}^2\ \right) $$

Using a separate set of weights ***w*** ^(1)^ and ***w*** ^(2)^ for each data type allows the model to assign the latent dimensions weights that differ between data types. We use the manifold relevance determination model implemented in GPy but constrain the model to use an ordinary RBF kernel rather than an ARD kernel. This model is thus equivalent to a shared GPLVM. The GPy implementation of manifold relevance determination uses a variational approximation to estimate the posterior, optimizing the evidence lower bound with respect to separate hyperparameters *σ* ^2^, *σ*_*rbj*_^2^, and *l* for each data type. We use the default initialization provided in GPy, which initializes the value of the latent space by performing PCA on the concatenated datasets.

### Learning warping functions

To learn warping functions from pseudotime to master time, we compute the sample quantiles of pseudotime for a specified number of quantiles, then align these sample quantiles with the theoretical quantiles of a uniform (0, 1) random variable. More precisely, we treat the sample quantiles of pseudotime as the independent values of an unobserved function and the theoretical quantiles of a uniform (0, 1) random variable as the dependent values of the function. Then we use either Gaussian process regression or linear interpolation to approximate the warping function that maps a pseudotime value to a master time value. We used 50 quantiles for all analyses in the manuscript, but found that the warping functions are robust to the number of quantiles used. Gaussian process regression is an attractive choice for learning a warping function due to the capability to capture non-linear effects and uncertainty, but Gaussian processes are not theoretically guaranteed to be monotonic. In practice, we found that the mean of the Gaussian process fit is monotonic in most cases, because the training data are monotonically increasing quantiles. For cases when the mean of the Gaussian process is not monotonic (as is the case for the single cell ChIP-seq data), we use linear interpolation. The monotonicity of the quantiles guarantees that the linear interpolation will be monotonic. The warping functions inferred for datasets in this study are shown in Additional file 1: Figure S7.

## Additional file

Additional file 1: Supplementary figures and legends. In this file, each legend links to the corresponding figure. (PDF 1350 kb)
