# Supplementary material for: MATCHER: manifold alignment reveals correspondence between single cell transcriptome and epigenome dynamics
Source: Genome Biol. 2017 Jul 24;18:138. doi: 10.1186/s13059-017-1269-0 (PMC5525279; doi:10.1186/s13059-017-1269-0)
Supplement: Additional file 1: — Supplementary figures and legends. In this file, each legend links to the corresponding figure. (PDF 1350 kb) [file 13059_2017_1269_MOESM1_ESM.pdf]

MATCHER: Additional file 1

Supplementary Figures

Supplementary Figure 1: MATCHER master time is strongly correlated with SLICER pseudotime. Scatterplot of SLICER pseudotime versus MATCHER master time for (a) RNA-seq, (b) bisulfite sequencing, (c) ATAC-seq, and (d) H3K4me2 ChIP-seq. The points are colored by SLICER pseudotime.

Supplementary Figure 2: Results from synthetic data generated from different underlying warping functions. Inferred warping functions for (a) linear, (b) square root, (c) quadratic, and (d) logit true underlying warping functions. (e)-(h) Scatterplot of true vs. inferred master time for the corresponding warp functions of panels (a)-(d).

Supplementary Figure 3: Synthetic data results for increasing noise levels.

Supplementary Figure 4: Corresponding values inferred by MATCHER for gene expression and chromatin accessibility signatures. Each point represents inferred correspondence from a single cell. The x-axis shows the value of the gene expression signature in that cell, and the y-axis shows the value of the chromatin accessibility signature. The points are colored by inferred master time. Note that these are the data used to generate the values on the diagonal of the heatmap in Fig. 4c.

Supplementary Figure 5: Corresponding values inferred by MATCHER for gene expression and H3K4me2 signatures. Each point represents inferred correspondence from a single cell. The x-axis shows the value of the gene expression signature in that cell, and the y-axis shows the value of the H3K4me2 signature. The points are colored by inferred master time. Note that these are the data used to generate the values on the diagonal of the heatmap in Fig. 4d.

Supplementary Figure 6: Subsampling analysis of sc-GEM data showing that MATCHER does not require corresponding cell measurements (a) Table of mean absolute deviation between ground truth and inferred correlations for scM&T-seq dataset (top row); scM&T-seq methylation data and Kolodziejczyk gene expression data (second row); the full sc-GEM dataset from Cheow; 5 random subsamples of 75% of cells from Cheow; and 5 random subsamples of 50% of cells from Cheow. (b)-(c) Density plots showing distribution of pseudotime inferred from (b) gene expression and (c) DNA methylation. The pseudotime values for individual cells are shown as a rug plot below the density plot; color indicates the time point. Compare panels (b)-(c) to Fig. 6 (a)-(b). (d) Violin plot of the DNA methylation master time values for cells at each time point. (e) Violin plot of the gene expression master time values for cells at each time point. Compare panels (d)-(e) to Fig. 6 (f)-(g).

Supplementary Figure 7: Inferred warping functions for all experimental datasets analyzed in the paper. (a) Kolodziejczyk single cell RNA-seq data, (b) Angermueller scM&T-seq methylation data, (c) ATAC-seq, (d) H3K4me2 ChIP data, (e) Angermueller scM&T-seq gene expression data, (f) warping function resulting from linear interpolation of H3K4me2 ChIP-seq data, (g) warping function for sc-GEM gene expression data, and (h) warping function for sc-GEM DNA methylation data.

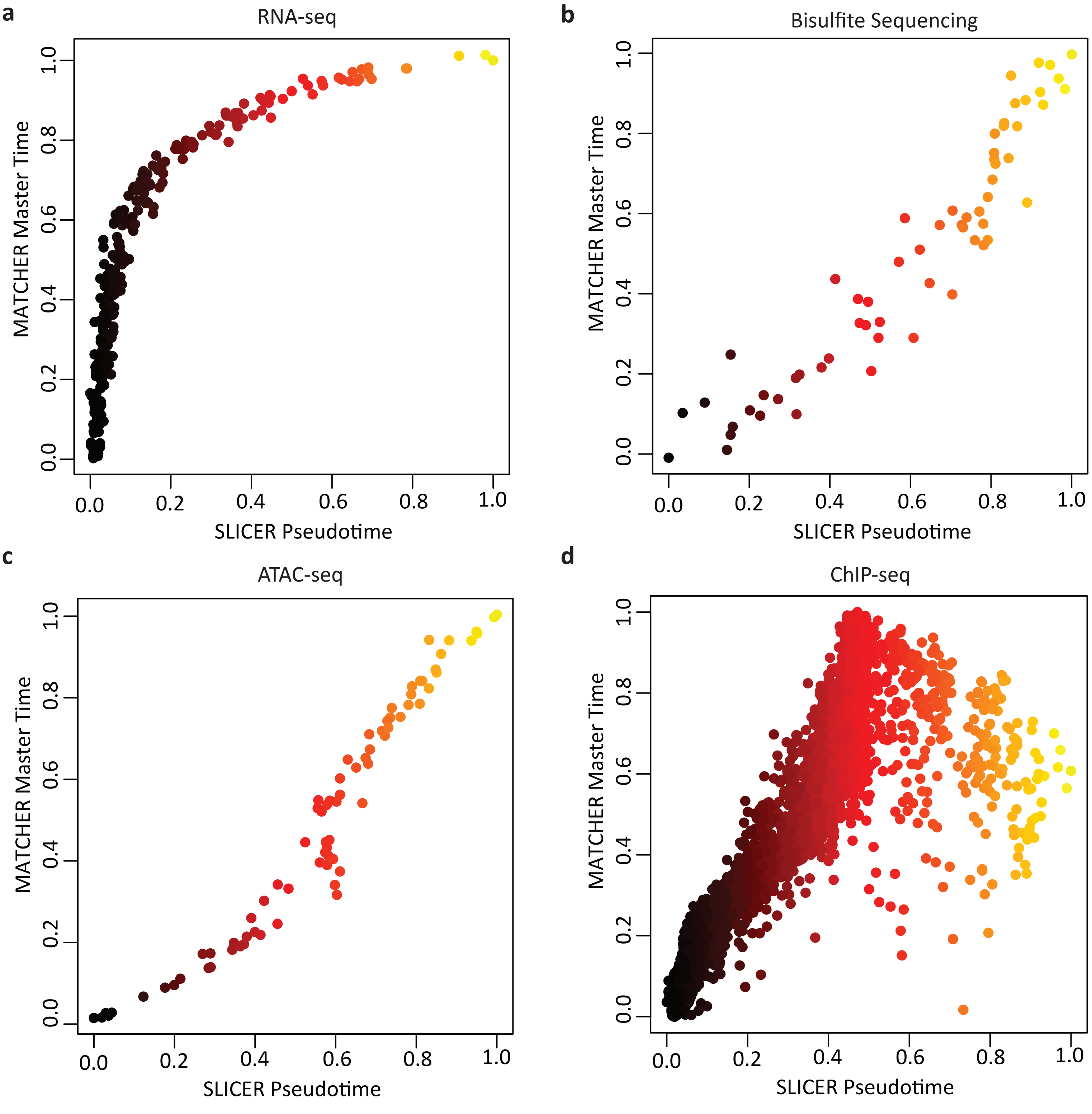

a
RNA-seq
b
Bisulfite Sequencing
c
ATAC-seq
d
ChIP-seq
MATCHER Master Time
SLICER Pseudotime
0.0
0.2
0.4
0.6
0.8
1.0

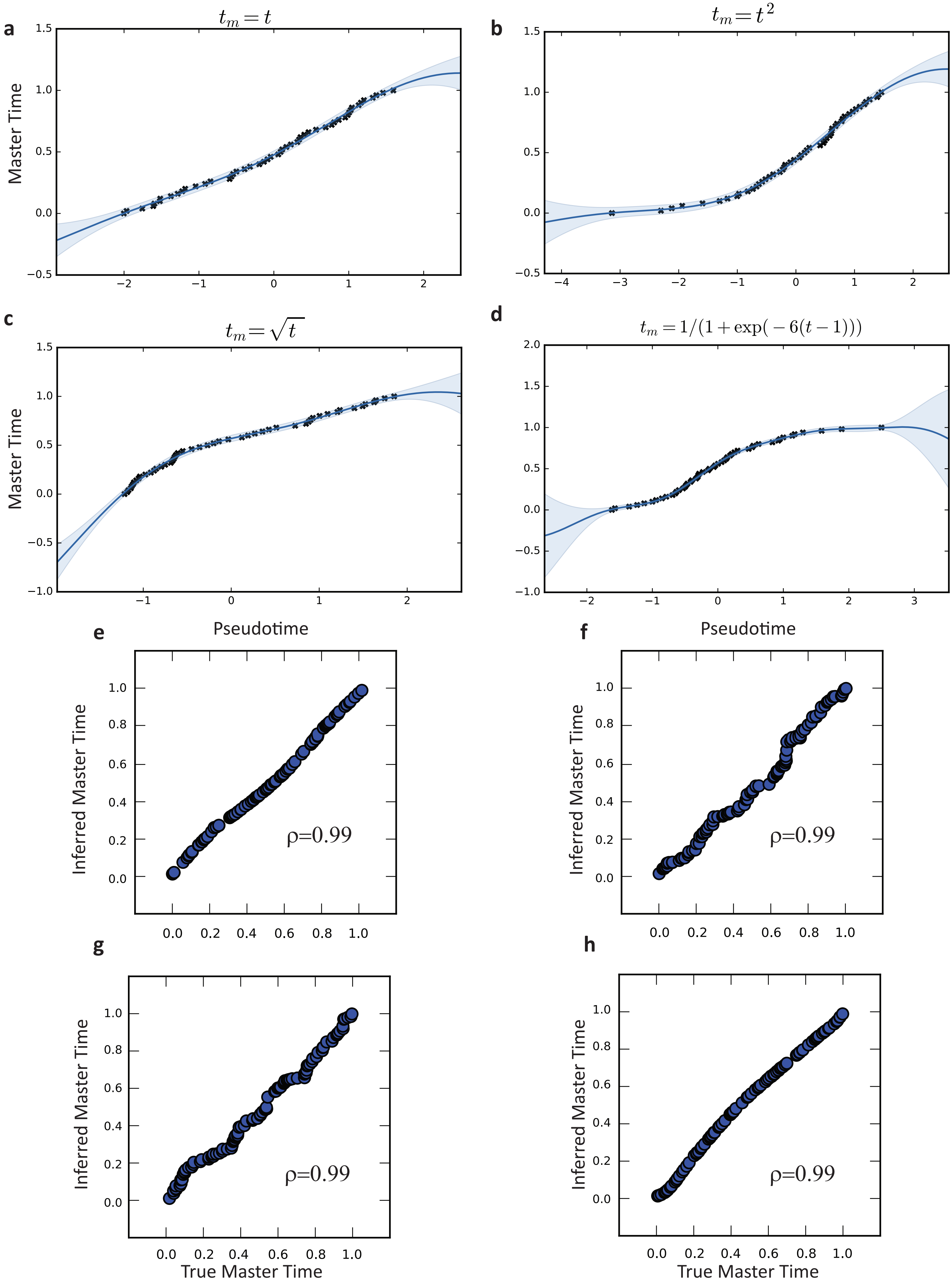
a
$t_m = t$
b
$t_m = t^2$
c
$t_m = \sqrt{t}$
d
$t_m = 1/(1+\exp(-6(t-1)))$
Master Time
Pseudotime
e
f
g
h
Inferred Master Time
True Master Time
ρ=0.99

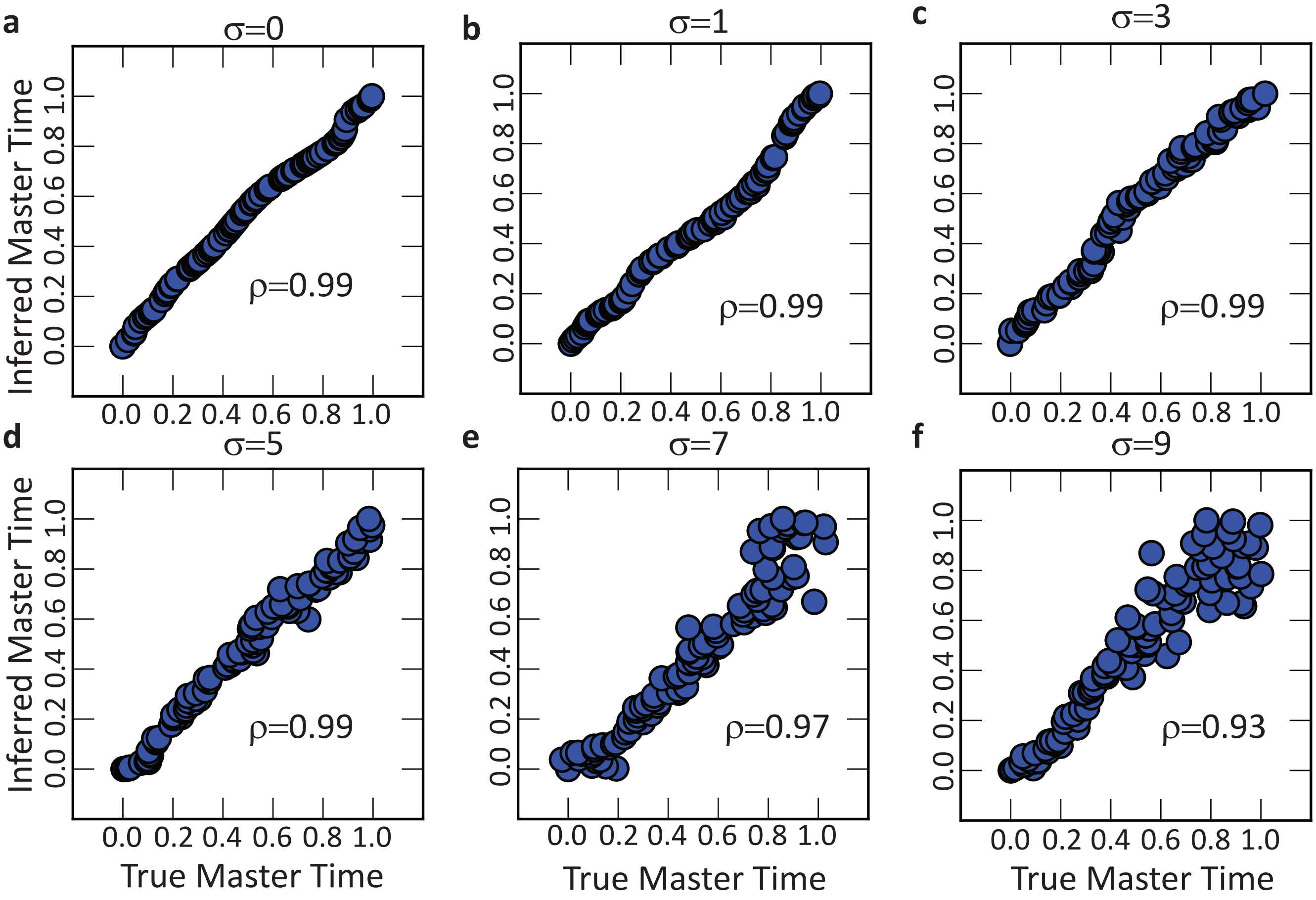

a
σ=0
ρ=0.99
b
σ=1
ρ=0.99
c
σ=3
ρ=0.99
d
σ=5
ρ=0.99
e
σ=7
ρ=0.97
f
σ=9
ρ=0.93
Inferred Master Time
True Master Time
0.0 0.2 0.4 0.6 0.8 1.0

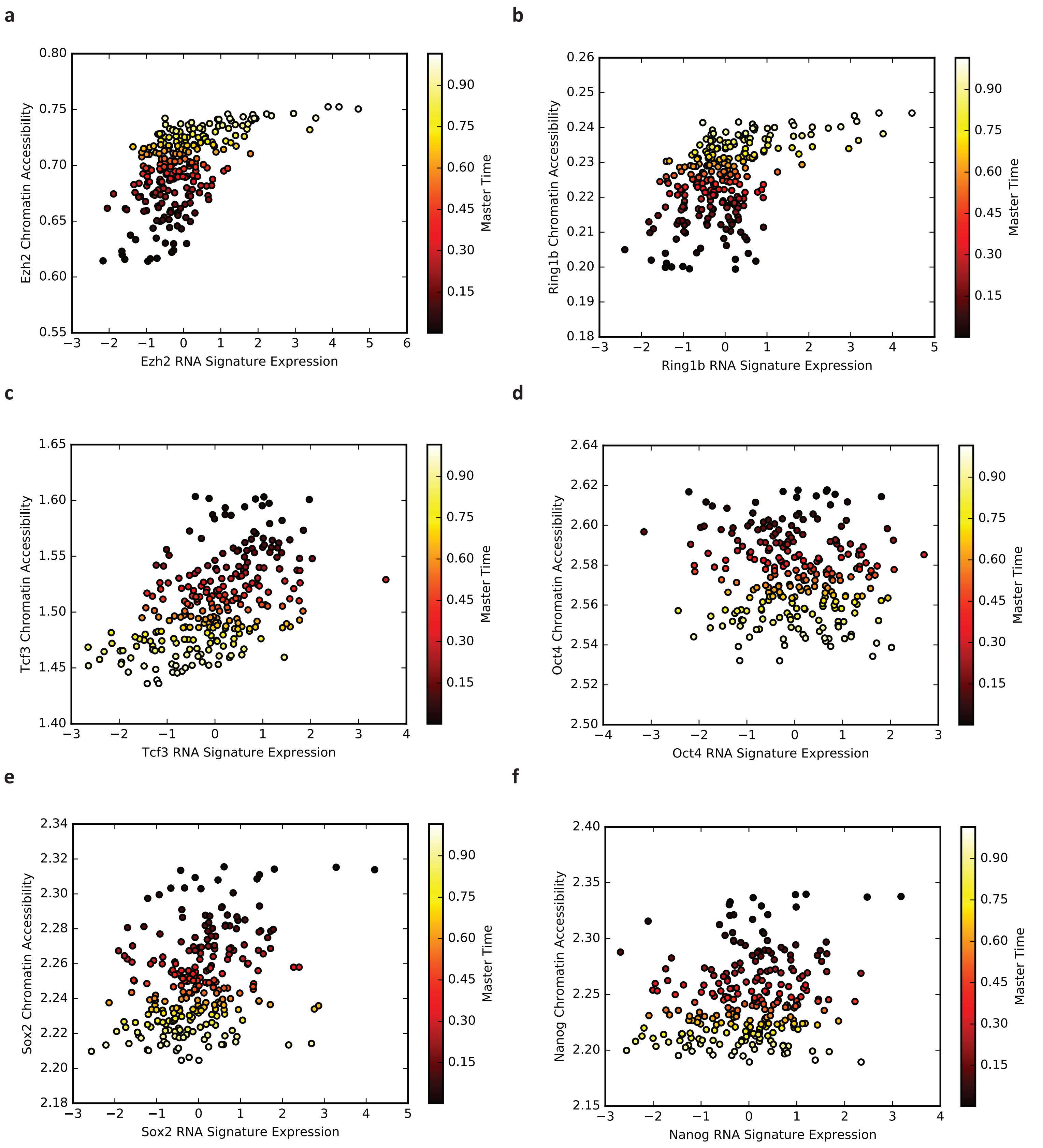
a
Ezh2 Chromatin Accessibility
Ezh2 RNA Signature Expression
Master Time
0.80
0.75
0.70
0.65
0.60
0.55
−3 −2 −1 0 1 2 3 4 5 6
0.90 0.75 0.60 0.45 0.30 0.15
b
Ring1b Chromatin Accessibility
Ring1b RNA Signature Expression
Master Time
0.26
0.25
0.24
0.23
0.22
0.21
0.20
0.19
0.18
−3 −2 −1 0 1 2 3 4 5
0.90 0.75 0.60 0.45 0.30 0.15
c
Tcf3 Chromatin Accessibility
Tcf3 RNA Signature Expression
Master Time
1.65
1.60
1.55
1.50
1.45
1.40
−3 −2 −1 0 1 2 3 4
0.90 0.75 0.60 0.45 0.30 0.15
d
Oct4 Chromatin Accessibility
Oct4 RNA Signature Expression
Master Time
2.64
2.62
2.60
2.58
2.56
2.54
2.52
2.50
−4 −3 −2 −1 0 1 2 3
0.90 0.75 0.60 0.45 0.30 0.15
e
Sox2 Chromatin Accessibility
Sox2 RNA Signature Expression
Master Time
2.34
2.32
2.30
2.28
2.26
2.24
2.22
2.20
2.18
−3 −2 −1 0 1 2 3 4 5
0.90 0.75 0.60 0.45 0.30 0.15
f
Nanog Chromatin Accessibility
Nanog RNA Signature Expression
Master Time
2.40
2.35
2.30
2.25
2.20
2.15
−3 −2 −1 0 1 2 3 4
0.90 0.75 0.60 0.45 0.30 0.15

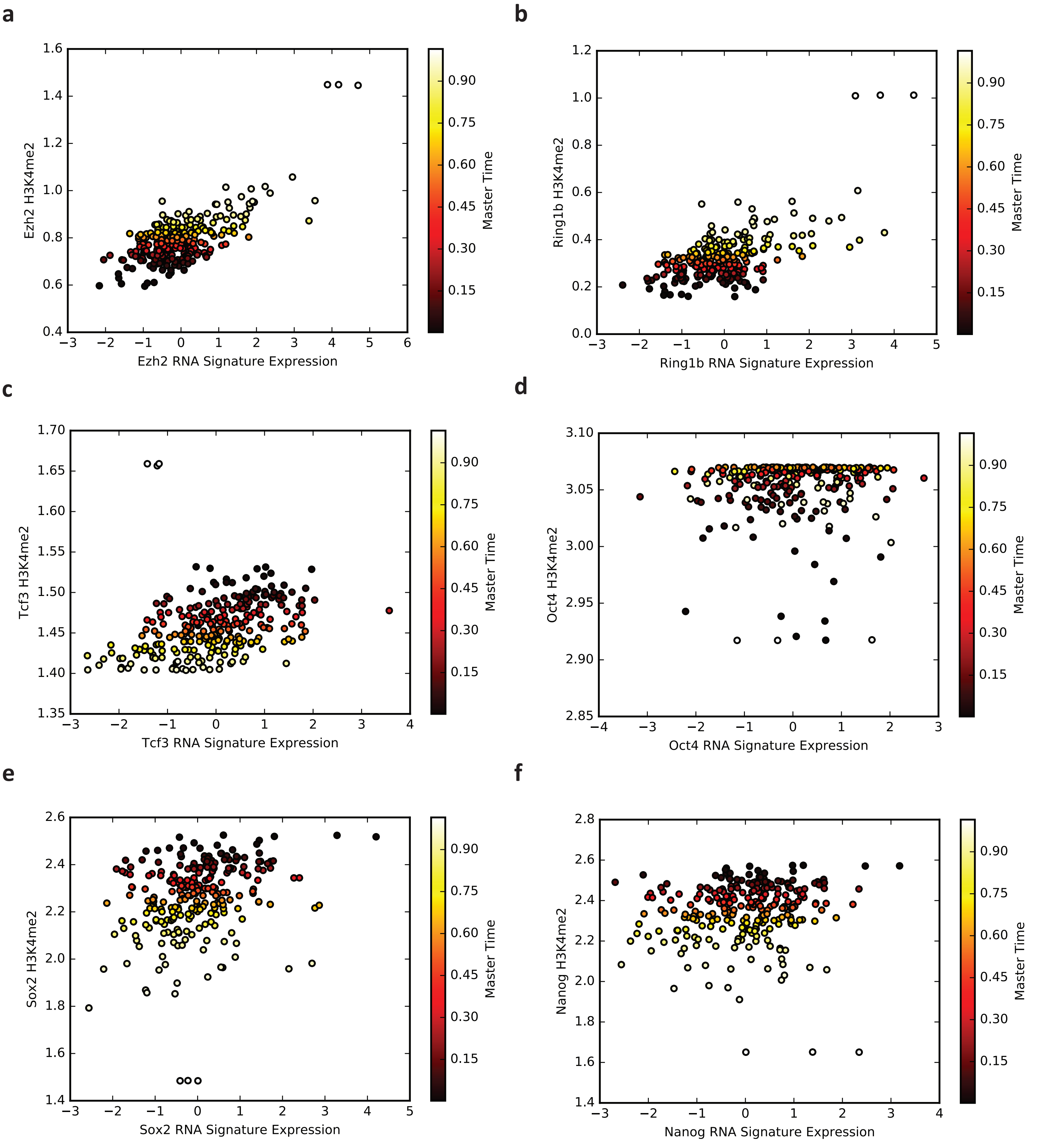
a
Ezh2 H3K4me2
Ezh2 RNA Signature Expression
Master Time
b
Ring1b H3K4me2
Ring1b RNA Signature Expression
Master Time
c
Tcf3 H3K4me2
Tcf3 RNA Signature Expression
Master Time
d
Oct4 H3K4me2
Oct4 RNA Signature Expression
Master Time
e
Sox2 H3K4me2
Sox2 RNA Signature Expression
Master Time
f
Nanog H3K4me2
Nanog RNA Signature Expression
Master Time

a

mESCs - scM&T	0.16				
mESCs - scRNA	0.27				
iPSCs - Full	0.17				
iPSCs - 75%	0.18	0.18	0.18	0.18	0.16
iPSCs - 50%	0.19	0.19	0.17	0.19	0.18

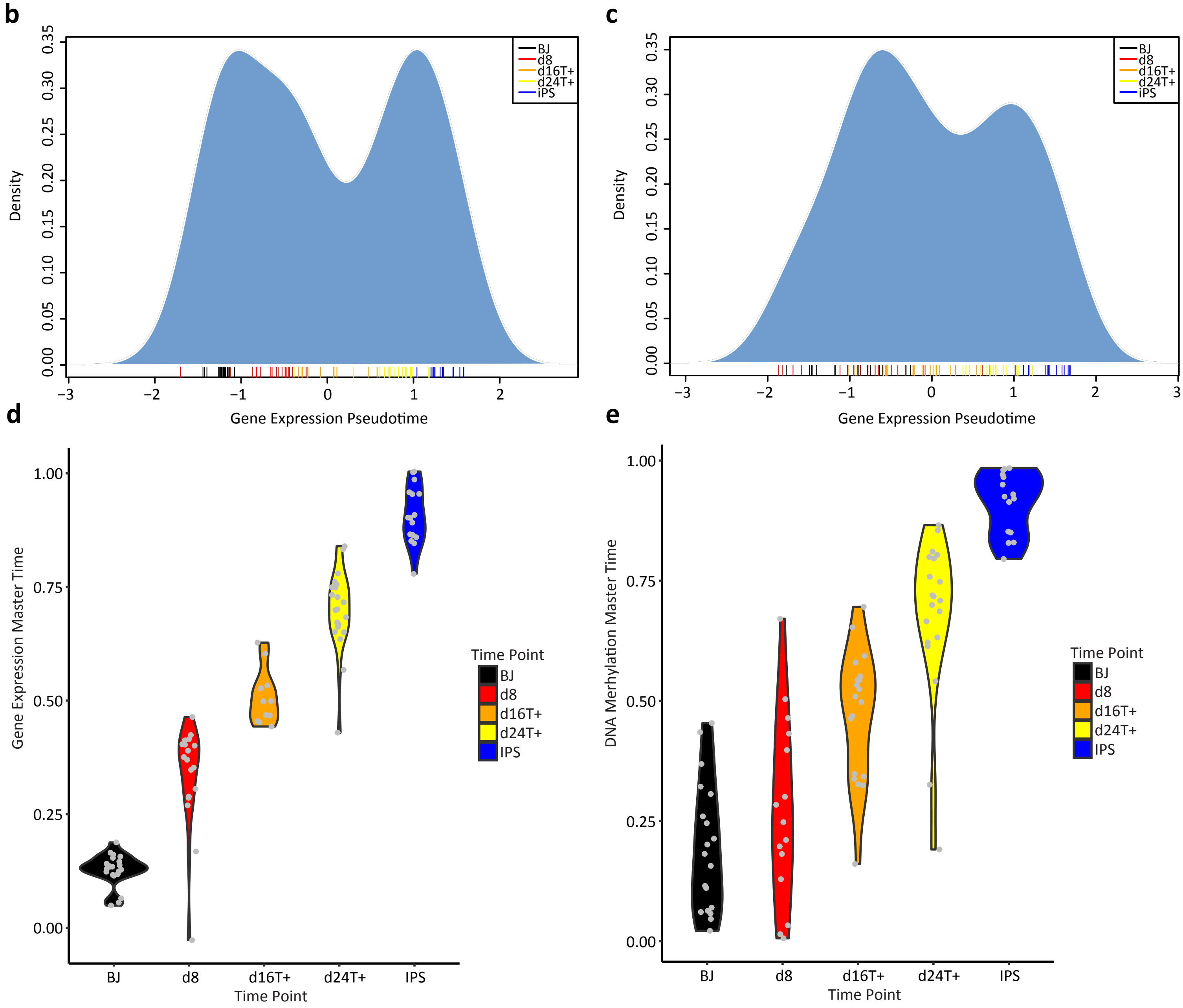

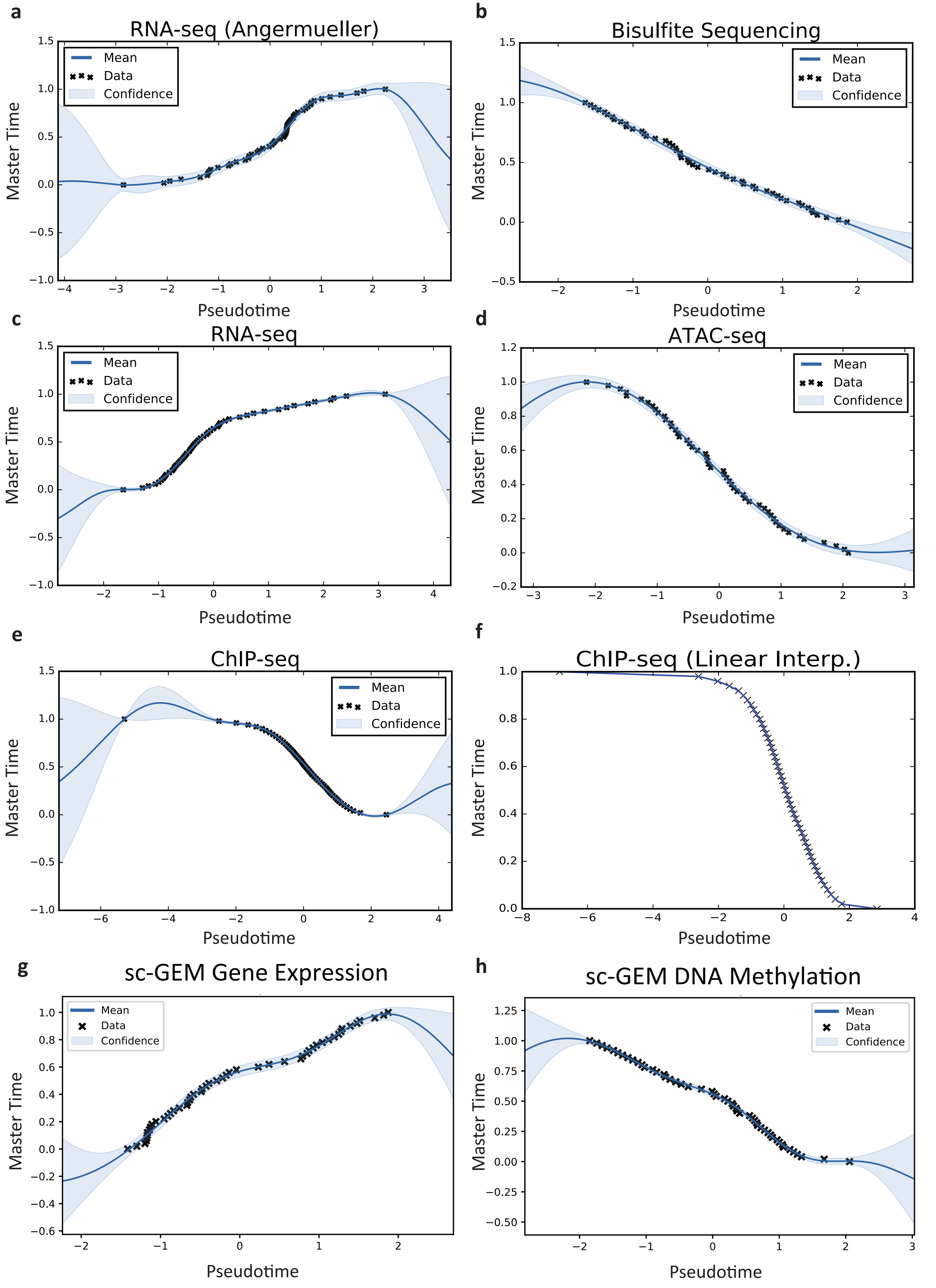

a
RNA-seq (Angermueller)
Mean
Data
Confidence
Master Time
Pseudotime
b
Bisulfite Sequencing
Mean
Data
Confidence
Master Time
Pseudotime
c
RNA-seq
Mean
Data
Confidence
Master Time
Pseudotime
d
ATAC-seq
Mean
Data
Confidence
Master Time
Pseudotime
e
ChIP-seq
Mean
Data
Confidence
Master Time
Pseudotime
f
ChIP-seq (Linear Interp.)
Master Time
Pseudotime
g
sc-GEM Gene Expression
Mean
Data
Confidence
Master Time
Pseudotime
h
sc-GEM DNA Methylation
Mean
Data
Confidence
Master Time
Pseudotime
